# The Mycobacterium tuberculosis sRNA F6 Modifies Expression of Essential Chaperonins, GroEL2 and GroES

**DOI:** 10.1128/Spectrum.01095-21

**Published:** 2021-09-22

**Authors:** Joanna Houghton, Angela Rodgers, Graham Rose, Alexandre D’Halluin, Terry Kipkorir, Declan Barker, Simon J. Waddell, Kristine B. Arnvig

**Affiliations:** a Faculty of Infectious Tropical Diseases, London School of Hygiene and Tropical medicine, London, United Kingdom; b Mycobacterial Metabolism and Antibiotic Research and Host-Pathogen Interactions in Tuberculosis Laboratories, The Francis Crick Institute, London, United Kingdom; c North Thames Genomic Laboratory Hub, Great Ormond Street Hospital for Children, London, United Kingdom; d Global Health and Infection, Brighton and Sussex Medical School, University of Sussex, Brighton, United Kingdom; e Structural and Molecular Biology, University College London, London, United Kingdom; University of Maryland School of Pharmacy

**Keywords:** *Mycobacterium tuberculosis*, small RNA, nutrient starvation, Wayne model chaperonins, infection, hypoxia, infection models, microarrays, persistence, small regulatory RNA

## Abstract

Almost 140 years after the identification of Mycobacterium tuberculosis as the etiological agent of tuberculosis, important aspects of its biology remain poorly described. Little is known about the role of posttranscriptional control of gene expression and RNA biology, including the role of most of the small RNAs (sRNAs) identified to date. We have carried out a detailed investigation of the M. tuberculosis sRNA F6 and shown it to be dependent on SigF for expression and significantly induced in starvation conditions *in vitro* and in a mouse model of infection. Further exploration of F6 using an *in vitro* starvation model of infection indicates that F6 affects the expression of the essential chaperonins GroEL2 and GroES. Our results point toward a role for F6 during periods of low metabolic activity typically associated with long-term survival of M. tuberculosis in human granulomas.

**IMPORTANCE** Control of gene expression via small regulatory RNAs (sRNAs) is poorly understood in one of the most successful pathogens, Mycobacterium tuberculosis. Here, we present an in-depth characterization of the sRNA F6, including its expression in different infection models and the differential gene expression observed upon deletion of the sRNA. Our results demonstrate that deletion of F6 leads to dysregulation of the two essential chaperonins GroEL2 and GroES and, moreover, indicate a role for F6 in the long-term survival and persistence of M. tuberculosis in the human host.

## INTRODUCTION

One hundred years after the launch of the Bacillus Calmette-Guerin (BCG) vaccine against tuberculosis (TB), this disease still claims more than 3,000 lives on a daily basis, and its etiological agent, Mycobacterium tuberculosis, remains one of the most prominent pathogens in human history. Notoriously difficult to work with and very different from both Gram-positive and Gram-negative model organisms, many aspects of its basic biology, including RNA biology and posttranscriptional control of gene expression, remain unclear. However, the study of regulatory RNA in M. tuberculosis is gaining momentum, aided by next-generation sequencing (NGS) applications, which have provided significant insights into the abundance and dynamics of noncoding RNA over a range of growth conditions, the location of transcription start sites on a global scale, and translated versus untranslated transcripts (1–5). Still, much remains to be uncovered about the posttranscriptional control exerted by regulatory RNA in M. tuberculosis, in particular what role these molecules play in the pathogenesis and persistence of M. tuberculosis. A multitude of M. tuberculosis small regulatory RNAs (sRNAs) have been identified in the last decade, but only few have been investigated, and even fewer have been linked to *M. tuberculosis* persistence and TB latency (1, 3, 4, 6–13).

F6 (ncRv10243) was one of the first M. tuberculosis sRNAs to be identified by cDNA cloning (6). F6 is conserved in a wide range of mycobacteria with the 5′ end showing the highest degree of conservation (6, 7). The location of F6 between the convergent *fadA2* (encoding an acetyl coenzyme A [acetyl-CoA] transferase) and *fadE5* (encoding an acyl-CoA hydrogenase) is also highly conserved, suggesting a potential role for F6 in the regulation of lipid metabolism, which is critical for intracellular survival (14). Initial analysis, combining Northern blotting with 5′ and 3′ rapid amplification of cDNA ends (RACE), revealed that F6 is expressed as a 102-nucleotide transcript, which is 3′ processed to the dominant transcript of 58 nucleotides (6). F6 is upregulated in the stationary phase, during oxidative stress, and by low pH, while overexpression from a multicopy-number plasmid leads to a slow-growth phenotype on solid media (6). The F6 promoter contains a typical SigF promoter motif and exhibits the highest SigF occupancy according to chromatin immunoprecipitation with microarray technology (ChIP-chip) analysis (6, 15). SigF is a nonessential sigma factor, conserved in most mycobacteria; its expression is induced by several stresses, including anaerobiosis, nutrient starvation, oxidative stress, and cold shock, while heat shock downregulates its expression, and deletion leads to attenuation in mice (16–19).

In the current study, we have investigated M. tuberculosis F6 expression under different growth conditions and found that this sRNA is significantly upregulated in a nutrient starvation model of persistence and in mouse lungs. We have generated an F6 deletion strain of M. tuberculosis and assessed the fitness of the Δ*f6* M. tuberculosis strain *in vitro* and *in vivo*. We found that inactivation of F6 leads to impaired recovery from a Wayne model of hypoxia. Moreover, using microarray analysis and reverse transcription-quantitative PCR (qRT-PCR), we have found that expression of the essential chaperonins encoded by *groEL2/rv0440* was significantly upregulated upon deletion of F6. Our results suggest that although F6 is highly upregulated in M. tuberculosis during the early stages of infection, it is likely to play a more prominent role during later stages of infection.

## RESULTS

### Expression of F6/SfdS is upregulated by starvation and during infection.

SigF is the predicted main regulator of F6 expression, and this was validated by Northern blotting, which demonstrated a complete absence of F6 in a Δ*sigF* strain (Fig. S1). We will henceforth refer to F6 as SfdS, for SigF-dependent sRNA. SigF is highly expressed during infection and in persister *in vitro* models such as nutrient starvation obtained by static incubation in phosphate-buffered saline (PBS) (16). We subjected M. tuberculosis H37Rv to starvation by washing and resuspending log-phase cultures in PBS for 24 and 96 h before isolating total RNA. SfdS expression was measured by qRT-PCR (normalized to 16S rRNA) and compared to log-phase levels.

**FIG 1:**
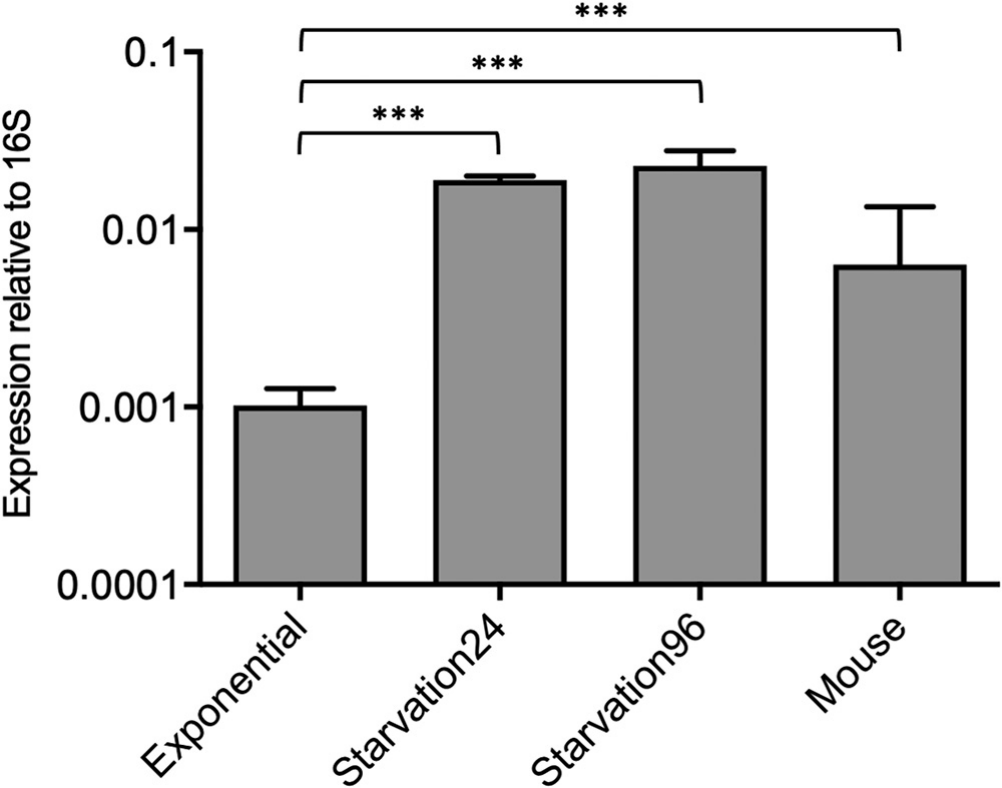
Expression of SfdS in M. tuberculosis. SfdS expression levels were measured in exponentially growing cultures, after 24 and 96 h in PBS (Starvation24 and Starvation96) and in mouse lungs after 3 weeks of infection using quantitative RT-PCT (qRT-PCR). Expression levels were normalized to 16S rRNA, and the data represent the mean and standard deviation of three biological replicates for each condition. ***, *P* value of <0.05 with significance tested using one-way analysis of variance (ANOVA).

The results demonstrate that SfdS expression increased dramatically (19-fold) within 24 h of starvation, with levels of SfdS remaining elevated for at least another 72 h (Fig. 1).

To investigate SfdS expression in a mouse model of infection, BALB/c mice were infected with ∼100 CFU of H37Rv via aerosol route and left for 3 weeks before culling and isolation of M. tuberculosis total RNA from lung tissue. We found that SfdS expression was robustly upregulated (∼6-fold) compared to log-phase levels but not to the same extent as that during *in vitro* starvation (Fig. 1).

Together, these results demonstrate that the expression of SfdS is dynamic and that it may play a role during infection, more specifically in nutrient-poor environments.

### Deletion of SfdS does not impair M. tuberculosis standard growth *in vitro*.

To identify a potential regulatory role of SfdS with minimal impact on the two flanking *fad* genes, we generated an unmarked SfdS deletion strain using site-directed mutagenesis and allelic exchange. Candidates were screened by PCR amplification and sequencing of the PCR product, which confirmed the deletion of the sRNA from the M. tuberculosis genome (not shown).

**FIG 2:**
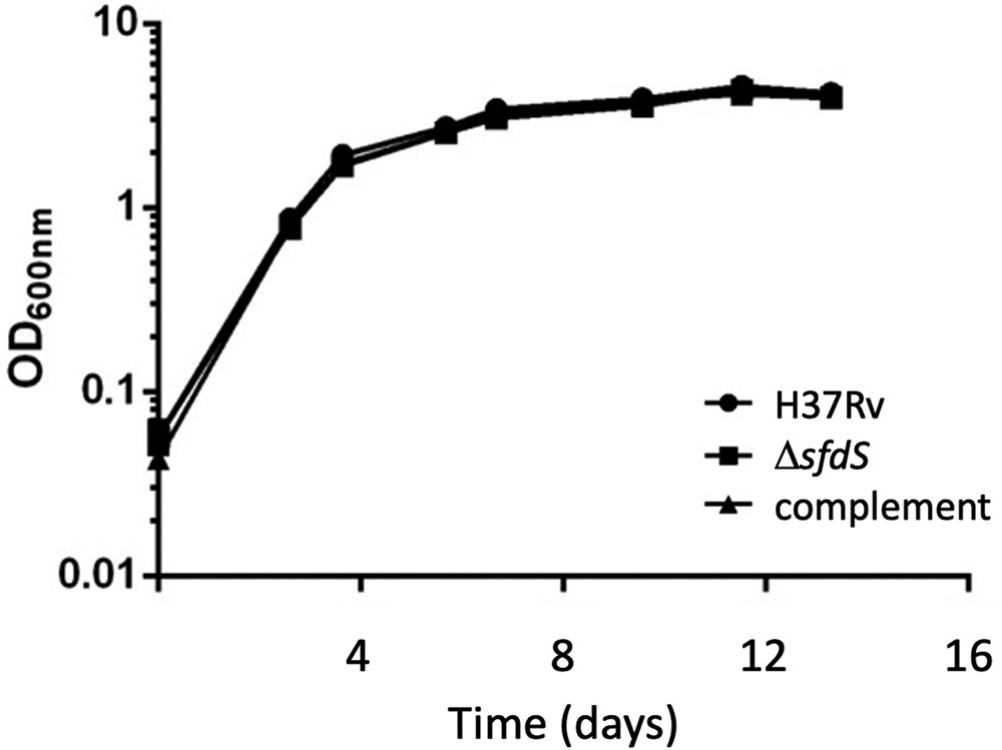
*In vitro* growth of Δ*sfdS*. Growth curves of wild-type M. tuberculosis H37Rv, Δ*sfdS*, and its complement in 7H9 in roller bottles. The results represent the average and standard deviation of three biological replicates.

To further verify the deletion and to determine if there were secondary mutations that might affect subsequent phenotypic analysis, wild-type and Δ*sfdS* strains were sequenced. Alignment of the genomic sequence of Δ*sfdS* with H37Rv identified two single-nucleotide polymorphisms (SNPs) in addition to the Xba I site deliberately introduced; one was C2864730T, resulting in an R102W substitution in Rv2541 (hypothetical protein), while the other was C4178146T in the 5′ untranslated region (UTR) of Rv3729 (potential transferase). To ensure that these SNPs did not influence the phenotype of the SfdS deletion strain, we constructed a complemented strain, in which expression of SfdS was driven by its native promoter from a single-copy plasmid integrated on the chromosome. Growth of wild-type H37Rv, Δ*sfdS*, and the complemented strain was monitored over a period of 2 weeks in 7H9 roller bottle cultures. Under these conditions, there was no significant difference between the three strains (Fig. 2).

**FIG 3:**
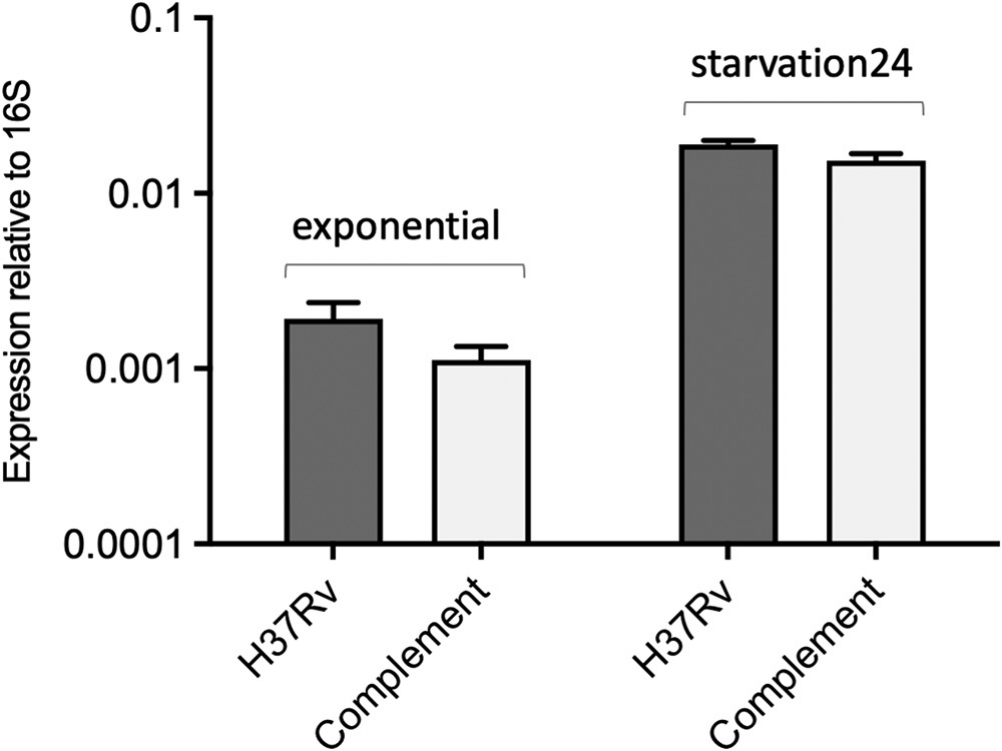
Expression of SfdS in wild-type H37Rv and complemented strain. RNA was isolated from log-phase and after 24 h starvation and analyzed by qRT-PCR. Data represent the mean and standard deviation of three biological replicates for each strain. Differences between wild-type and complement are not significant according to unpaired *t* test.

To validate deletion and complementation, expression of SfdS was measured in wild-type H37Rv, Δ*sfdS*, and the complemented strain during exponential growth and after 24 h of PBS starvation, using qRT-PCR. The results demonstrated that there was no detectable expression of SfdS in the deletion strain (Fig. S2) and that there was a small, nonsignificant difference between SfdS expression in wild-type and complemented strains in both growth conditions (unpaired *t* test, *P* < 0.05, Fig. 3).

### Fitness of the Δ*sfdS* strain in macrophages and mouse models of infection.

**FIG 4:**
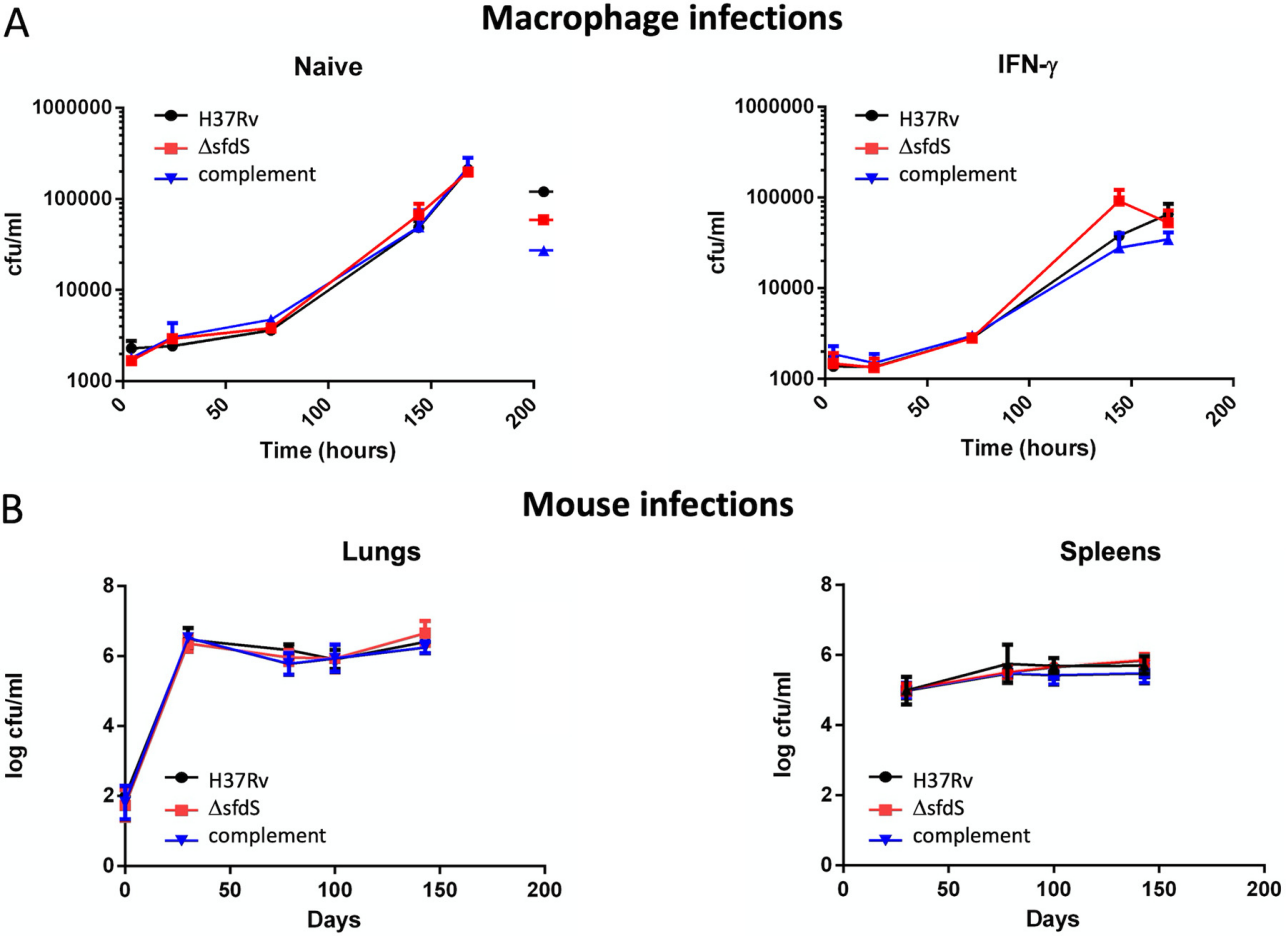
Survival of the Δ*sfdS* in models of infection. Panel A shows survival of wild-type M. tuberculosis H37Rv, Δ*sfdS*, and complementing strains in a macrophage model of infection with naive (left) and IFN-γ activated (right) BMDM. Data represent the mean and standard deviation of triplicate infections. Statistical significance was tested with one-way ANOVA, *P* < 0.05. Panel B shows survival of the three strains within the lungs and spleens of BALB/c mice. Data represent the averages and standard deviations from 5 mice per time point. Statistical significance was tested with two-way ANOVA.

As we had observed a robust upregulation of SfdS during infection, we wanted to compare the fitness of wild-type M. tuberculosis H37Rv, Δ*sfdS*, and the complemented strain during infection. The three strains were used to infect naive and interferon gamma (IFN-γ) preactivated murine bone marrow-derived macrophages (BMDMs) at a multiplicity of infection (MOI) of 0.1:1 (bacteria/macrophages). The infection was allowed to continue for 7 days with five time points. At each time point, the macrophages were lysed and plated for CFU. For both naive and activated macrophages, we observed no significant differences between the three strains, suggesting that under these conditions, deletion of SfdS does not result in attenuation (Fig. 4A).

A macrophage model of infection does not entirely replicate the conditions encountered by the bacteria in more complex animal models of infection. To assess the role of SfdS in pathogenesis in a more representative infection model, wild-type M. tuberculosis H37Rv, Δ*sfdS*, and the complemented strain were compared in a mouse model of infection. BALB/c mice were infected with approximately 100 CFU via the aerosol route, and the infection was followed for 143 days. On days 0, 30, 78, 100, and 143, lungs and spleens were harvested for bacterial enumeration. Again, no significant difference was observed in CFU between the wild-type M. tuberculosis H37Rv and Δ*sfdS* at any of the time points (Fig. 4B).

### Recovery from Wayne model hypoxia is impaired in Δ*sfdS*.

**FIG 5:**
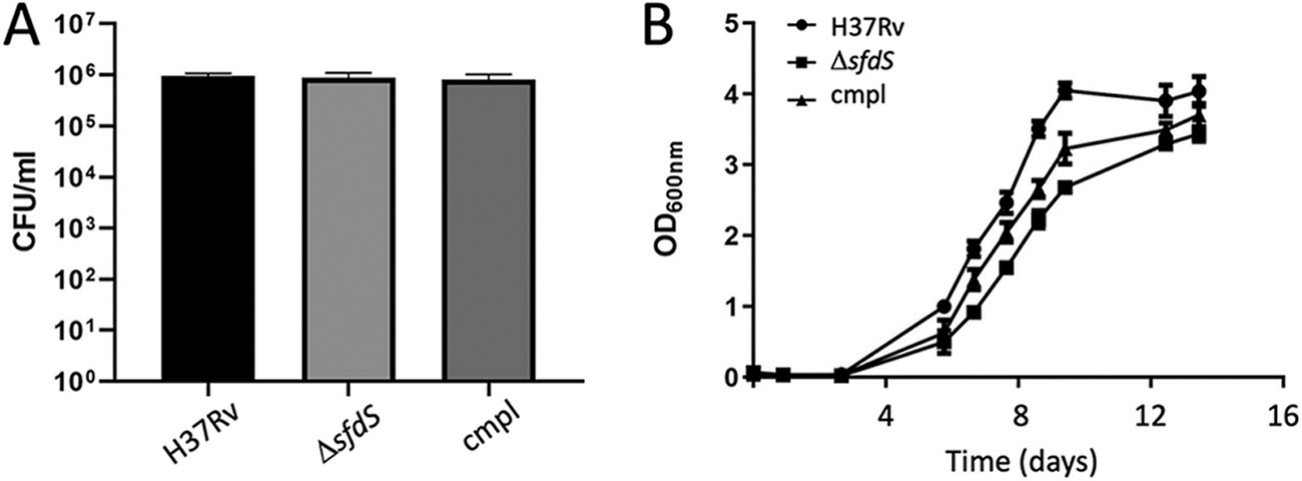
Recovery from NRP-2 is impaired in Δ*sfdS*. Wild-type M. tuberculosis H37Rv, Δ*sfdS*, and complement (cmpl) strains were grown in the Wayne model until NRP-2 was reached. Cultures were diluted and plated for CFU counts. (A) Number of viable bacteria after incubation in the Wayne model. (B) All cultures were subsequently adjusted to an OD_600_ of 0.05 in fresh media, and growth was measured over 14 days. All data represent the averages and standard deviation of three biological replicates. Paired *t* test between wild-type and Δ*sfdS* as well as between Δ*sfdS* and complement indicated a significant difference (*P* < 0.05) in postrecovery growth.

One of the hallmarks of human TB is the formation of granulomas, which are absent in a standard mouse model. Granulomas are characterized by limited nutrient and oxygen availability, among other things (20). A widely used *in vitro* model of hypoxia is the Wayne model, in which the available oxygen is gradually limited in sealed cultures of M. tuberculosis (21). When oxygen concentrations decrease to the microaerobic level (1% oxygen saturation), the cells enter a state of nonreplicating persistence, NRP-1, followed by NRP-2 when oxygen saturation reaches 0.06%. SigF is highly induced during anaerobiosis, suggesting that its regulon, including SfdS, may play a role in low-oxygen conditions (19). We therefore decided to evaluate the fitness of the Δ*sfdS* strain using the Wayne model. To measure potential differences in respiration rate, the depletion of oxygen for each strain was monitored by a methylene blue indicator tube set up for each strain at the start of incubation. After 14 days of incubation, at NRP-2, cultures were plated onto 7H11 agar for determination of CFU. In addition, NRP-2 cells were assessed for their ability to be resuscitated by dilution into fresh 7H9 media and monitoring of growth (by optical density at 600 nm [OD_600_]). There was no significant difference in survival (CFU) between wild-type H37Rv, Δ*sfdS*, and the complemented M. tuberculosis strain at day 14 incubation in the Wayne model (Fig. 5A). Conversely, the regrowth of NRP-2 cells transferred to fresh media indicated that the Δ*sfdS* strain was impaired for recovery/resuscitation following incubation in the Wayne model, while the complemented strain displayed an intermediate phenotype (Fig. 5B). These results suggest that SfdS may play a role in the resuscitation of nonreplicating M. tuberculosis, which is associated with the reactivation of latent infection.

### Deletion of SfdS leads to upregulation of essential chaperonins.

To establish a potential regulatory role for SfdS, we employed nutrient starvation, as this was the most potent inducer of the sRNA in our hands. RNA was isolated from triplicate cultures of wild-type H37Rv and Δ*sfdS* starved for 24 h in PBS and analyzed using Agilent microarrays.

**FIG 6:**
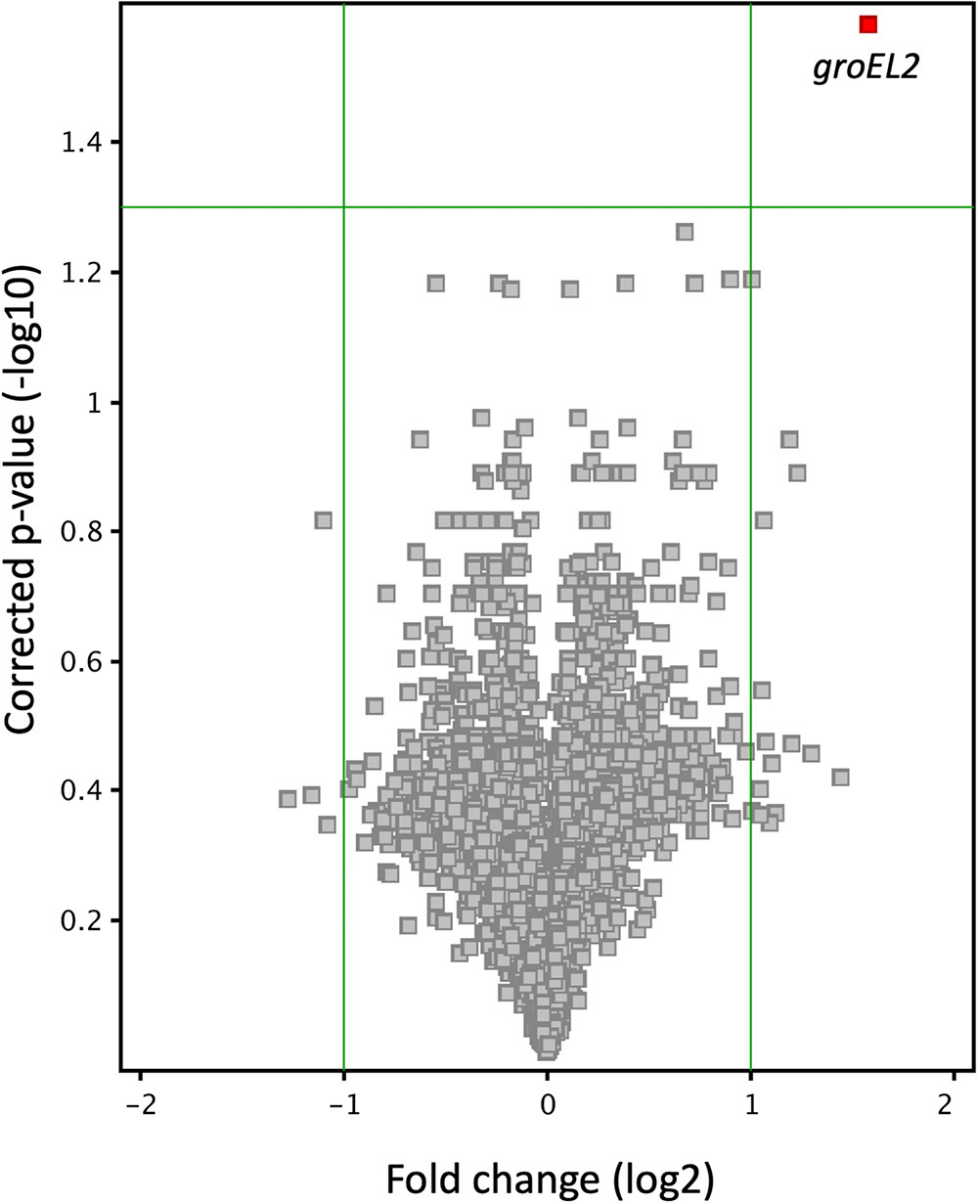
Volcano plot of Δ*sfdS* versus H37Rv upon starvation in PBS. The plot shows *groEL2* as the only gene whose expression level was significantly changed (≥2-fold differentially expressed) between Δ*sfdS* and H37Rv.

Strikingly, only a single gene, *groEL2/rv0440*, was significantly differentially expressed, with a 3-fold upregulation in the Δ*sfdS* strain compared to that in wild-type H37Rv (Fig. 6). However, with a less-stringent analysis, i.e., removing the multiple testing correction but maintaining the cutoff at a 2-fold change, we observed nine differentially expressed genes, of which five (*dnaK*, *groEL2*, *rv0990c*, *rv2012*, and *rv3224*) are associated with heat shock in M. tuberculosis (Fig. S3) (22).

**FIG 7:**
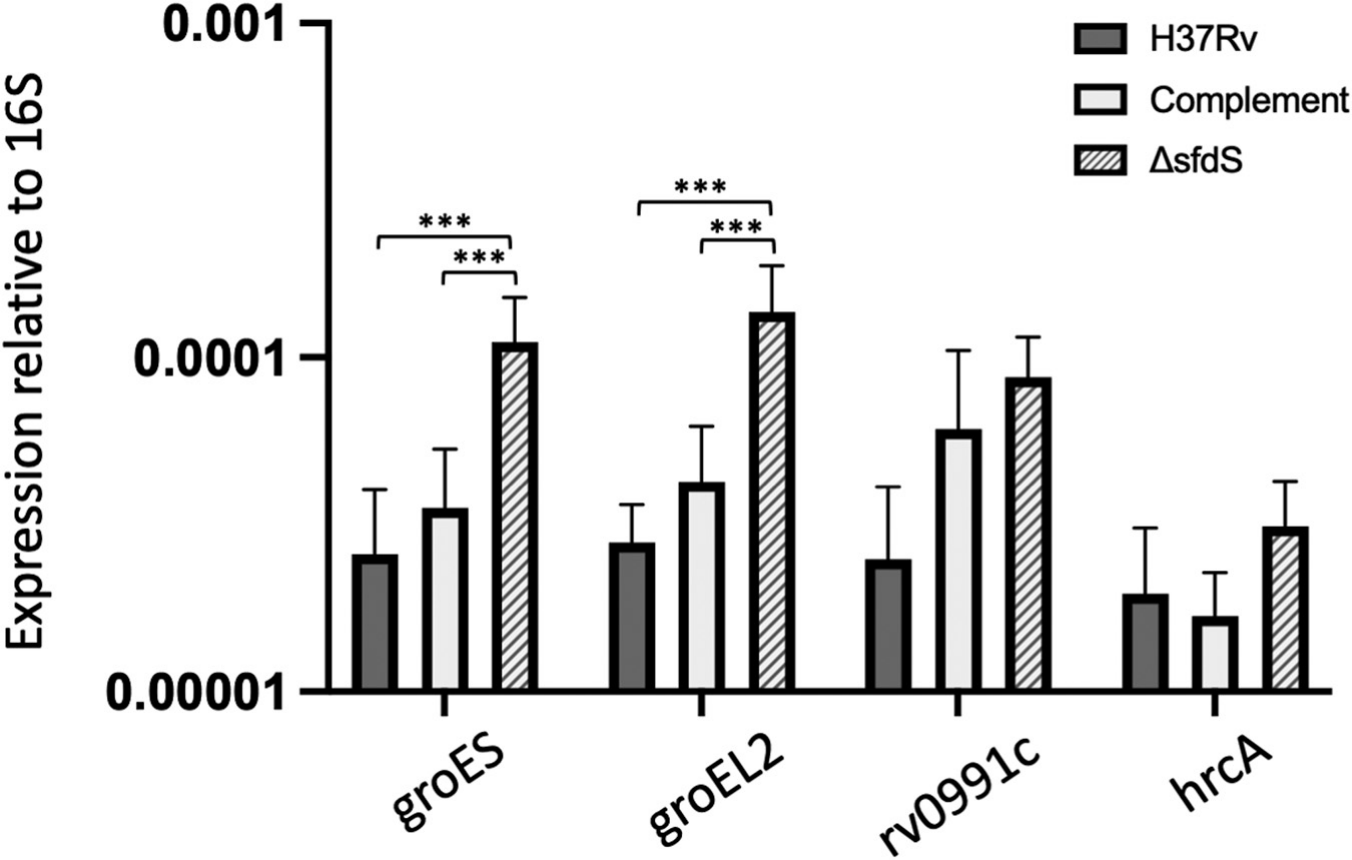
Expression of HrcA-regulated genes in *M. tuberculosis* strains. qRT-PCR was performed on total RNA from M. tuberculosis H37Rv (wild-type), Δ*sfdS* mutant, and its complement. Each bar shows the expression level of the indicated genes normalized to 16S rRNA. All data represent the mean and standard deviation of three biological replicates for each strain. ***, *P* value of <0.05 with significance tested using one-way ANOVA.

In an effort to identify potential, direct mRNA targets of SfdS, we used the TargetRNA2 webserver with default settings and full-length SfdS against the nine differentially expressed genes (23). However, no direct targets could be predicted in this way. Instead, we assumed that the observed changes represent downstream effects and hypothesized that the differentially expressed genes had a common regulator. Four of the nine genes (*dnaK*, *groEL2*, *rv0990c*, and *rv3224*) are specifically associated with HrcA, which directly regulates transcription of *groEL2*, the *groES-groEL1* and *rv0991c-rv0990c* operons, and its own expression, i.e., the *hrcA-dnaJ2* operon (22, 24). To ensure that expression of *groEL2* was restored to wild-type levels in the complemented strain, we performed qRT-PCR on RNA extracted from three biological replicates of wild-type H37Rv, Δ*sfdS*, and the complemented strain. The results confirmed that *groEL2* was significantly upregulated in Δ*sfdS* upon starvation and that the phenotype could be complemented by providing a copy of the *sfdS* gene in *trans* (Fig. 7). To probe if the observed changes in *groEL2* expression could be associated with transcriptional control by HrcA, we also measured the expression of the first gene in each of the HrcA-controlled operons, i.e., *groES*, *rv0991c*, and *hrcA*, in the three M. tuberculosis strains. Similar to the expression of *groEL2*, that of *groES* was significantly upregulated in Δ*sfdS* and restored in the complemented strain; *rv0991c* and *hrcA* displayed similar trends, but the changes were not statistically significant, and therefore the role of HrcA remains inconclusive (Fig. 7).

## DISCUSSION

The M. tuberculosis SigF-controlled sRNA SfdS (F6) is well expressed in the exponential phase but upregulated by a variety of stresses (6).

In this study, we have shown that SfdS is also highly upregulated after 3 weeks of murine infection and, to an even higher extent, in the PBS starvation model at 24 and 96 h. This suggests that SfdS plays a role in the early stages of pathogenesis but possibly a more significant one for survival in nutrient-deficient environments, such as those encountered in human granulomas in later stages of infection. M. tuberculosis may resuscitate after periods of low metabolic activity (21, 25, 26), and using the Wayne model of nonreplicating persistence, we found that recovery of Δ*sfdS* from NRP-2 was impaired compared to wild-type and complemented strains, although initial survival was unchanged. The fact that the complemented strain displayed an intermediate phenotype may be due to *cis*-regulatory effects associated with the deletion, the different genomic location of the complementing SfdS, or the point mutations identified with the whole-genome sequencing (WGS).

Our results indicate that starvation for 24 h leads to the upregulation of HrcA-controlled chaperonins *groES* and *groEL2* in the Δ*sfdS* strain compared to the expression in the wild-type and complemented M. tuberculosis strains, while the expression of the remaining HrcA-regulated operons (*rv0991c-0990c* and *hrcA-dnaJ2*) was not significantly changed. However, regulation of HrcA expression and activity is highly complex and the lack of a significant change of *hrcA* mRNA levels in the Δ*sfdS* strain does not exclude a role for this regulator in the observed response.

Prediction of potential mRNA targets of SfdS using the TargetRNA2 webserver (23) did not result in any direct targets, and we conclude that the observed changes likely represent downstream effects from other, as yet unidentified targets.

Nevertheless, the fact that SfdS appears to repress chaperonin expression does offer a potential explanation for the previously observed slow growth associated with SfdS overexpression (6), namely, an untimely repression of these essential chaperonins, which could lead to reduced protein synthesis. This also suggests that the overexpression strain may be hypersensitive to heat shock.

RNA is a ubiquitous temperature-sensing molecule, and several heat shock response mechanisms employ RNA, including the eukaryotic heat shock transcription factor 1 (HSF1), Escherichia coli *rpoH*, and Deinococcus radiodurans sRNA DnrH, the latter of which activates the expression of Hsp20 (27–29). Further analysis using pulsed rather than constitutive overexpression or deletion may provide more clues on the link between SfdS and heat shock.

Infection of BMDMs for 7 days with H37Rv, Δ*sfds*, and complement revealed no attenuation of the deletion strain. Similarly, we observed no difference in the CFU recovered from the lungs and spleens of BALB/c mice after 143 days of infection. As SfdS expression is not only directed by SigF but also dominant in terms of promoter occupancy, one might expect some parallels between infection with Δ*sfdS* and Δ*sigF*. Indeed, a lack of attenuation has been observed in a study of a Δ*sigF* strain in human monocyte derived macrophages, while late stages of infection and time-to-death experiments result in attenuation of a CDC1551 Δ*sigF* strain (18, 30). Given that starvation is a potent inducer of SigF/SfdS expression, it seems possible that a more long-term infection and/or the ability to form granulomas is required to observe strong phenotypes associated with the deletion of SfdS. Alternatively, a higher MOI might change the course of infection. Moreover, the mouse model of infection does not replicate all aspects of an M. tuberculosis infection in humans, one key difference being the lack of granuloma formation in a standard mouse model such as BALB/c (31). Granulomas provide a unique niche for M. tuberculosis in which oxygen and nutrients are limited, the latter being the strongest inducer of SfdS expression in our study. A different animal model, e.g., C3HeB/FeJ mice, which develop necrotic lung granulomas, may provide more answers (32). Our results point toward a role for SfdS during periods of low metabolic activity similar to those of cold shock and may be associated with nutrient starvation conditions, such as those found in human granulomas in later stages of infection, and it remains a possibility that SfdS plays a role in surviving and resuscitating from this environment.

## MATERIALS AND METHODS

### Bacterial strains and plasmids.

Escherichia coli DH5α was used for plasmid construction and grown in LB agar or broth using kanamycin at 50 μg/ml and X-Gal where necessary at 200 μg/ml. Mycobacterium tuberculosis H37Rv was grown on Middlebrook 7H11 agar plus 10% oleic acid-albumin-dextrose-catalase (OADC) (Becton, Dickinson). Liquid cultures were grown in standard Middlebrook 7H9 medium supplemented with 0.5% glycerol, 10% Middlebrook ADC (Becton, Dickinson), and 0.05% Tween 80 at 37°C in a roller bottle (Nalgene) rolling at 2 rpm or 50-ml falcon tubes (Corning) in an SB3 tube rotator (Stewart) at 28 rpm. Kanamycin was added where required at 25 μg/ml and X-Gal where required at 50 μg/ml. All plasmids and oligonucleotides used in this study are listed in Tables S1 and S2.

### The Wayne model.

Cultures were grown to exponential phase (OD_600_ = 0.6 to 0.8) and subsequently diluted to an OD_600_ of 0.005 in 7H9 in triplicate in Wayne tubes, each containing a sterile stirring bar. Cultures were incubated at 37°C on a stirring platform, and OD was monitored over time until cultures reached NRP-2. Cultures were then either diluted into 7H9, OD_600_ 0.05, and OD monitored or serially diluted onto 7H11 agar and CFU enumerated.

### Construction of F6/SfdS deletion and complemented strains.

Allelic replacement techniques were used to generate an M. tuberculosis knockout mutant as per published protocol (33). Briefly, approximately 1.5 kb of the flanking region from either side of F6/SfdS (5′ flank coordinates 292520 to 293605 and 3′ flank 293709 to 294600) was cloned into the suicide vector pBackbone (34). The sRNA was replaced by an Xba I site using site-directed mutagenesis to produce the targeting plasmid pJHP04. Electrocompetent H37Rv was transformed with pJHP04, and subsequent single crossovers (SCOs) and double crossovers (DCOs) were selected (see supplemental material for details).

To complement the Δ*f6/sfdS* mutant, electrocompetent cells were prepared and transformed with an integrating plasmid (pJHP06) containing the cloned region 293428 to 293876 from M. tuberculosis H37Rv and supplemented with pBSInt, providing the phage integrase (35). Complementation was confirmed using qRT-PCR.

### Mapping genome data and variant calling.

To determine the genome sequence of the deletion strain, WGS was performed as described elsewhere using the Illumina HiSeq platform (2). Sequencing reads for the Δ*f6/sfdS* strain were mapped against the M. tuberculosis H37Rv reference genome (AL123456) using Burrows-Wheeler aligner (BWA) (36), and variants were called with SAMtools (37). Variant filtering was performed by inclusion of only those variants with a minimum mapping quality of 10 and maximum read depth of 400. Finally, heterozygous calls or those found in repetitive or mobile elements (genes annotated as PE/PPE/insertions/phages) were removed.

### Preparation of starved cultures.

M. tuberculosis H37Rv, Δ*f6/sfdS*, and complemented strain were grown in Middlebrook 7H9 supplemented with 0.4% glycerol, 0.085% NaCl, 0.5% bovine serum albumin (BSA), and 0.05% tyloxapol in roller bottle culture (2 rpm at 37°C). Exponentially growing bacteria were pelleted and washed 3 times with PBS supplemented with 0.025% tyloxapol and finally resuspended in triplicate in PBS with 0.025% tyloxapol to an equivalent starting volume and incubated statically for 24 or 96 h.

### RNA isolation.

RNA isolation from *in vitro* cultures was done as described previously (6). Briefly, we harvested cultures with rapid cooling by adding ice directly to the culture and subsequent centrifugation at 10,000 rpm for 10 min. RNA was isolated from the pellet using the FastRNA pro blue kit from MP Biomedicals following the manufacturer’s instructions.

To isolate RNA from bacteria grown in mice, lung homogenates were spun at 13,000 rpm for 5 min to collect the bacteria. These were resuspended in 1 ml TRIzol (Invitrogen) with 150-μm glass beads, and the samples were disrupted in a FastPrep (MP Biomedicals) at a setting of 6.0 for 40 s. The RNA was extracted according to the manufacturer’s guidelines. RNA concentration was measured by Nanodrop (Thermo Scientific), and RNA integrity was measured by the 2100 Bioanalyzer using a nano chip (Agilent Technologies).

### Northern blotting.

Ten micrograms each of H37Rv and Δ*sigF* total RNA was separated on a 10% denaturing acrylamide gel and transferred onto Brightstar-Plus nylon membrane (Ambion) by electroblotting. RNA was UV crosslinked to the membrane and stained with 0.3 M sodium acetate/0.03% methylene blue to verify transfer. 32P-labeled riboprobes were synthesized using the mirVana probe construction kit (Ambion) and 32P-UTP (800 mCi/mmol, PerkinElmer) with the template oligonucleotide listed in Table S2 and hybridized to the membranes overnight in UltraHyb (Invitrogen).

### Quantitative RT-PCR.

Total RNA was treated with Turbo DNase (Ambion) until DNA free. cDNA was synthesized using Superscript III (Invitrogen) and random hexamers. Primers were designed using the Applied Biosystems software Primer Express, and sequences are listed in Table S1. Each 20-μl qRT-PCR contained 16SYBRgreen (Applied Biosystems), 900 nM each primer, and 5 μl of template cDNA. Absolute quantitation was carried out, and all genes were normalized to 16S rRNA expression.

### Transcriptional profiling.

Whole-genome M. tuberculosis microarray slides were purchased from Agilent Technologies through the Bacterial Microarray Group at St. George’s (BμG@S), University of London. For cDNA synthesis, 2 μg wild-type H37Rv and Δ*sfdS* knockout isolated from 24-h starved cultures was used. The cDNA was labeled individually with both Cy-3 and Cy-5 dyes (GE Healthcare) using Superscript III reverse transcriptase (Invitrogen). Dye swaps were performed, and the cDNA was hybridized to an 8 Chamber Agilent slide at 65°C for 16 h before slide was washed with Oligo aCGH wash buffer 1 (Agilent) for 5 min at room temperature and Oligo aCGH wash buffer 2 (Agilent) for 1 min at 37°C. Slides were stabilized using Agilent stabilization and drying solution according to the manufacturer’s instructions.

Slides were scanned at 5 μm using an Agilent Technologies microarray scanner at BμG@S. Txt files created by the Agilent scanner were analyzed using Genespring 14.5 filtering on flags and expression. *t* test against zero was performed using a *P* value of <0.05 with Benjamini-Hochberg multiple testing correction and 2-fold cutoff. Array design is available in ArrayExpress (accession no. A-BUGS-41). Microarray data have been deposited into ArrayExpress (accession number E-MTAB-9327).

### F6 target prediction.

Prediction of potential F6 sRNA targets was performed using the TargetRNA2 webserver (http://cs.wellesley.edu/~btjaden/TargetRNA2/index.html) with differentially expressed genes and default parameters (23).

### Macrophage infection.

Bone marrow-derived macrophages (BMDMs) were generated from 6- to 8-week-old BALB/c mice in RPMI 1640 (Gibco) containing 10% fetal calf serum, 20 μM l-glutamine, 1 mM sodium pyruvate, 10 μM HEPES, and 50 nM β-mercaptoethanol. The cells were then grown and differentiated in complete RPMI 1640 supplemented with 20% L929 cell supernatant for 6 days at 37°C in 5% CO_2_. The differentiated cells were seeded at a density of 2 × 10^5^ cells/well in 1 ml complete RPMI 1640 supplemented with 5% L929 cell supernatant and incubated overnight prior to infection. M. tuberculosis strains for infection were grown to an OD_600_ of 0.5 to 0.8, and inocula were prepared by washing and resuspending the cultures in PBS to produce a single cell suspension. This was used to infect BMDMs at a multiplicity of infection (MOI) of 0.1:1. After 4 h, the cells were washed to remove all extracellular bacilli, medium was replaced, and incubation was continued. Macrophages were lysed with water-0.05% Tween 80 to release intracellular bacteria after 4, 24, 72, 120, and 168 h postinfection. Bacilli were serially diluted in PBS-Tween and plated on 7H11 with OADC. Plates were incubated for 3 to 4 weeks for CFU counts. All experiments were performed in triplicate.

### Murine infection model and ethics statement.

Groups of 6- to 8-week-old BALB/c mice were infected by low-dose aerosol exposure with M. tuberculosis H37Rv wild type, Δ*f6/sfdS*, and the complemented strain using a Glas-Col (Terre Haute, IN) aerosol generator calibrated to deliver approximately 100 bacteria into the lungs. Bacterial counts in the lungs (*n* = 5) at each time point of the study were determined by plating serial dilutions of individual lung homogenates on duplicate plates of Middlebrook 7H11 agar containing OADC enrichment. CFU were counted after 3 to 4 weeks incubation at 37°C. BALB/c mice were bred and housed under specific pathogen-free conditions at the Medical Research Council, National Institute for Medical Research (NIMR). All mouse studies and breeding were approved by the animal ethics committee at NIMR. Protocols for experiments were performed under project license number 80/2236, in accordance with Home Office (United Kingdom) requirements and the Animal Scientific Procedures Act, 1986.

### Data availability.

Array design is available in ArrayExpress (accession no. A-BUGS-41). Microarray data have been deposited in ArrayExpress (accession number E-MTAB-9327).
