# Supplementary material for: The Mycobacterium tuberculosis sRNA F6 Modifies Expression of Essential Chaperonins, GroEL2 and GroES
Source: Microbiol Spectr. 2021 Sep 22;9(2):e01095-21. doi: 10.1128/Spectrum.01095-21 (PMC8557902; doi:10.1128/Spectrum.01095-21)
Supplement: SUPPLEMENTAL FILE 1 — Supplemental material. Download SPECTRUM01095-21_Supp_1_seq11.pdf, PDF file, 1.2 MB [file spectrum01095-21_supp_1_seq11.pdf]

SUPPLEMENTARY FIGURES 1-3

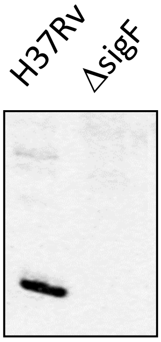

Fig. S1: No F6 expression in Δ*sigF* mutant. 10 μg each of H37Rv and Δ*sigF* total RNA was separated on a 10% denaturing acrylamide gel and transferred onto Brightstar-Plus nylon membrane (Ambion) by electroblotting. RNA was UV cross-linked to the membrane and stained with 0.3 M sodium acetate/0.03% methylene blue to verify transfer. 32P-labelled riboprobes were synthesised using the mirVana Probe construction Kit (Ambion) and 32P-UTP (800 mCi/mmol, PerkinElmer) with the template oligo listed Table S2, and hybridised to the membranes overnight in UltraHyb (Invitrogen).

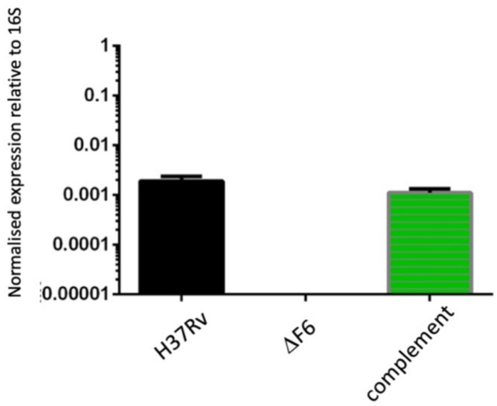

Fig. S2 Expression of F6 in different strains

RNA from 7H9 cultures of H37Rv, ΔF6 and the complemented strain was harvested in exponential phase and F6 levels measured by qRT-PCR normalised to 16S rRNA. Data represents the average and standard deviation of three biological replicates for each strain.

Statistical Testing – t-test against zero p<0.05 (no MTS) – 2 fold cutoff

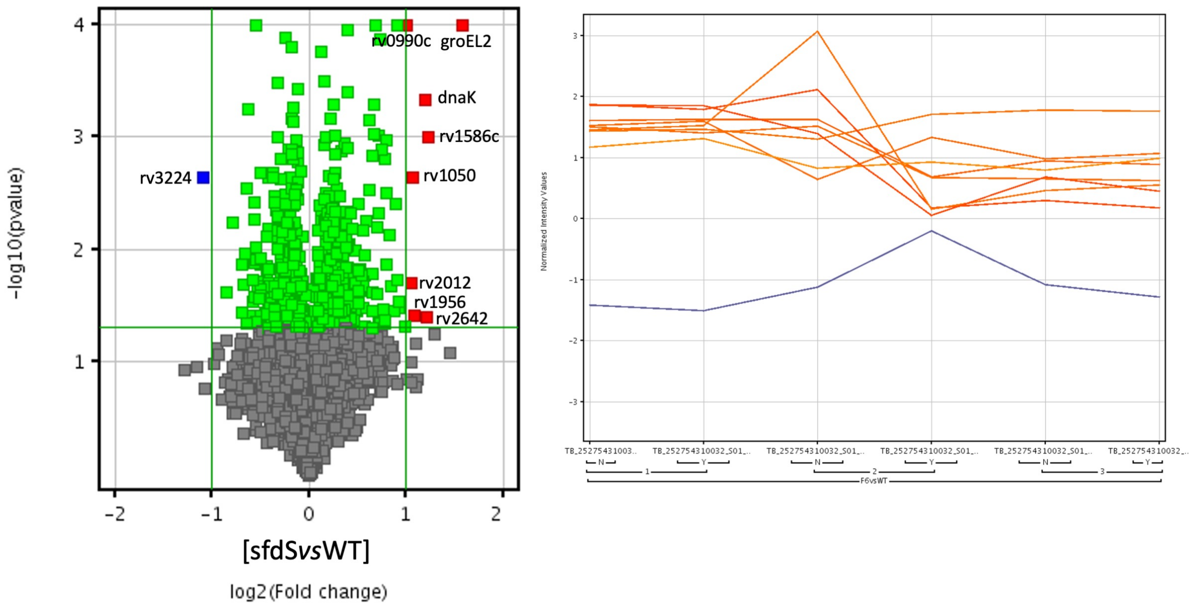

Fig. S3 Differential gene expression without multiple testing correction. Volcano plot illustrating nine genes were differentially expressed when using less stringent statistical testing, i.e. data was not subjected to multiple testing correction.
