# Supplementary material for: The Mycobacterium tuberculosis sRNA F6 Modifies Expression of Essential Chaperonins, GroEL2 and GroES
Source: Microbiol Spectr. 2021 Sep 22;9(2):e01095-21. doi: 10.1128/Spectrum.01095-21 (PMC8557902; doi:10.1128/Spectrum.01095-21)
Supplement: SUPPLEMENTAL FILE 2 — Supplemental material. Download SPECTRUM01095-21_Supp_2_seq12.pdf, PDF file, 0.1 MB [file spectrum01095-21_supp_2_seq12.pdf]

SUPPLEMENTARY TABLES S1-S2

Table S1: Plasmids used in this study

Plasmid	Relevant Characteristic	Source or Reference
pBackbone	Mycobacterial suicide vector (kanamycin^R and ampicillin^R)	(1)
pKP186	Integrating mycobacterial cloning vector that does not contain intergrase (kanamycin^R)	(2)
PBSInt	Mycobacterial suicide vector containing integrase, electroporated in conjunction with pKP186 and its derivatives.	(3)
pJHP04	Targeting plasmid for removal of F6 in *M. tuberculosis*. pBackbone containing F6 5' and 3' flanking regions and the *sacB/lacZ* cassette	This Study
pJHP06	F6 complementing plasmid. pKP186 derivative containing 448bp coordinates 293428-293876	This Study

Table S2: Oligos used in this study

Name	Sequence (5'-3')	Description
F6RevXbaI	GGTCTAGACGAGTGATCGGG	F6 targeting plasmid
F6ForXbaI	GGTCTAGATGGGCTTGCCC	F6 targeting plasmid
F6RevSDM	GGGGCAAGCCCAAAAAGATCTAGACCGAGTGATC GGGTACCC	F6 targeting plasmid
F6ForSDM	GGGTACCCGATCACTCGGTCTAGATCTTTTTGGGC TTGCCCC	F6 targeting plasmid
F6compF	GAAAAGCTTGCCGCTGTTGACCAG	F6 complement plasmid
F6compR	CGGATCCCTGCGCGGGCTGA	F6 complement plasmid
F6nrt	CGGATAGCCCCGTGTTGTTGTCTGACCTGTCTC	F6 Northern probe template
16sTqmF	TCCCGGGCCTTGTACACA	qRT-PCR
16sTqmR	CCACTGGCTTCGGGTGTTA	qRT-PCR
F6TqmF	GGATAGCCCCGTGTTGTTG	qRT-PCR
F6TqmR	GGGATTGCCCCGCATT	qRT-PCR
Rv0440TqmF	CGTCGTCCTGGAAAAGAAGTG	qRT-PCR
Rv0440TqmR	GGTCTTCTTGGCTACCTCTTTGAC	qRT-PCR

Rv3418cTqmF	CGTTGCGGAGGGTGACA	qRT-PCR
Rv3418cTqmR	TCCTCGCCGTTGTACTTGATC	qRT-PCR
Rv0990cTqmF	GGCCGCGCACGATCT	qRT-PCR
Rv0990cTqmR	CGTTTTTCCAGCCTGACATCA	qRT-PCR
Rv0991cTqmF	TTCAAAGGCACCGGCTTCTA	qRT-PCR
Rv0991cTqmR	TGGTCTGGCTCTTGGACTTCTT	qRT-PCR

References for supplementary data

1. Gopaul KK, Brooks PC, Prost JF, Davis EO. 2003. Characterization of the two Mycobacterium tuberculosis recA promoters. J Bacteriol 185:6005-15.
2. Rickman L, Scott C, Hunt DM, Hutchinson T, Menendez MC, Whalan R, Hinds J, Colston MJ, Green J, Buxton RS. 2005. A member of the cAMP receptor protein family of transcription regulators in Mycobacterium tuberculosis is required for virulence in mice and controls transcription of the rpfA gene coding for a resuscitation promoting factor. Mol Microbiol 56:1274-86.
3. Springer B, Master S, Sander P, Zahrt T, McFalone M, Song J, Papavinasasundaram KG, Colston MJ, Boettger E, Deretic V. 2001. Silencing of oxidative stress response in Mycobacterium tuberculosis: expression patterns of ahpC in virulent and avirulent strains and effect of ahpC inactivation. Infect Immun 69:5967-73.
